# Recent advances in recombinant protein-based malaria vaccines

**DOI:** 10.1016/j.vaccine.2015.09.093

**Published:** 2015-12-22

**Authors:** Simon J. Draper, Evelina Angov, Toshihiro Horii, Louis H. Miller, Prakash Srinivasan, Michael Theisen, Sumi Biswas

**Affiliations:** a The Jenner Institute, University of Oxford, Old Road Campus Research Building, Roosevelt Drive, Headington, Oxford OX3 7DQ, UK; b Walter Reed Army Institute of Research, U. S. Military Malaria Research Program, Malaria Vaccine Branch, 503 Robert Grant Avenue, Silver Spring, MD 20910, USA; c Department of Molecular Protozoology, Research Institute for Microbial Diseases, Osaka University, 3-1 Yamadaoka, Suita, Osaka 561-873, Japan; d Malaria Cell Biology Section, Laboratory of Malaria and Vector Research, National Institutes of Allergy and Infectious Diseases, National Institutes of Health, Rockville, MD 20852, USA; e Department for Congenital Disorders, Statens Serum Institut, Copenhagen, Denmark; f Centre for Medical Parasitology at Department of International Health, Immunology, and Microbiology and Department of Infectious Diseases, Rigshospitalet, University of Copenhagen, Copenhagen, Denmark

**Keywords:** Malaria, Recombinant protein, Vaccine, Adjuvant, *Plasmodium falciparum*, Antibody

## Abstract

*Plasmodium* parasites are the causative agent of human malaria, and the development of a highly effective vaccine against infection, disease and transmission remains a key priority. It is widely established that multiple stages of the parasite's complex lifecycle within the human host and mosquito vector are susceptible to vaccine-induced antibodies. The mainstay approach to antibody induction by subunit vaccination has been the delivery of protein antigen formulated in adjuvant. Extensive efforts have been made in this endeavor with respect to malaria vaccine development, especially with regard to target antigen discovery, protein expression platforms, adjuvant testing, and development of soluble and virus-like particle (VLP) delivery platforms. The breadth of approaches to protein-based vaccines is continuing to expand as innovative new concepts in next-generation subunit design are explored, with the prospects for the development of a highly effective multi-component/multi-stage/multi-antigen formulation seeming ever more likely. This review will focus on recent progress in protein vaccine design, development and/or clinical testing for a number of leading malaria antigens from the sporozoite-, merozoite- and sexual-stages of the parasite's lifecycle–including PfCelTOS, PfMSP1, PfAMA1, PfRH5, PfSERA5, PfGLURP, PfMSP3, Pfs48/45 and Pfs25. Future prospects and challenges for the development, production, human delivery and assessment of protein-based malaria vaccines are discussed.

## Highlights

• Protein-based vaccines remain the cornerstone approach for B cell and antibody induction against leading target malaria antigens.
• Advances in antigen selection, immunogen design and epitope-focusing are advancing the field.
• New heterologous expression platforms are enabling cGMP production of next-generation protein vaccines.
• Next-generation antigens, protein-based immunogens and virus-like particle (VLP) delivery platforms are in clinical development.
• Protein-based vaccines will form part of a highly effective multi-component/multi-stage/multi-antigen subunit formulation against malaria.

## 1 Introduction

Multiple stages of the *Plasmodium* parasite's lifecycle are susceptible to antibodies, including the liver-invasive sporozoite; the red blood cell (RBC)-invading merozoite; parasite stages within the infected erythrocyte (iRBC) which display antigen at the cell surface; as well as the sexual-stage forms present in both the human host and mosquito vector. This susceptibility has led to myriad efforts to develop subunit vaccines that can induce functional antibodies capable of preventing malaria infection, disease or transmission [1].

All subunit vaccines in their most basic form require delivery of antigen(s) believed to be targets of protective immunity, coupled with an immuno-stimulant or ‘adjuvant’ selected in the belief that this will lead to the induction of a strong and durable immune response of the appropriate type. Even these most basic of tenets have proved challenging in the context of antibody-inducing subunit vaccines for malaria, but much progress has been made.

The classical approach to antibody induction by subunit vaccination has been the delivery of protein antigen formulated in adjuvant, with notable success in humans including examples such as hepatitis B virus surface antigen (HBsAg) and bacterial toxoids (tetanus and diphtheria). In the case of malaria, the production of conformational recombinant proteins using heterologous expression platforms can prove challenging, especially when using bacterial-based systems [2]. However, numerous protein vaccine candidates have now been successfully produced to current Good Manufacturing Practice (cGMP) standard (using amongst others *Escherichia coli*, *Saccharomyces cerevisiae* and *Pichia pastoris*), and progressed to Phase I/II clinical testing (Table 1). Clinical progression also requires access to adjuvants which are safe and display acceptable reactogenicity profiles [3], but in turn are capable of eliciting antibody responses of sufficient magnitude to prevent rapidly invading or developing parasites. Our incomplete understanding of protective immune effector mechanisms *in vivo* in humans also continues to hamper vaccine development and prioritization [4]. Whether antibodies function through cell-independent neutralization type mechanisms or *via* Fc-mediated immune cell interactions is often unclear, as is the potential contribution of CD4^+^ T helper cell responses to B cell induction and memory maintenance, and IgG affinity maturation and subtype polarization. How adjuvant selection and antigen delivery can skew these parameters in humans is still poorly understood. With regard to antigen target selection, the malaria parasite genome possesses over 5000 genes, with complex expression patterns throughout all stages of the lifecycle [5]. The historical absence of biological information on the vast majority of gene products has meant that subunit vaccine development has traditionally focused on a relatively limited number of well-studied candidates.

Despite these great challenges, huge progress has been made with recombinant protein malaria subunits. Numerous antigens and adjuvants have now been tested in Phase I/II clinical trials yielding important and informative clinical data (Table 1). A variety of expression platforms have been used to produce soluble proteins, fusion antigens, long synthetic peptides (LSP), conjugates and antigen arrayed on virus-like particles (VLPs). Indeed, the leading anti-sporozoite subunit vaccine, RTS,S/AS01B, based on a recombinant VLP of HBsAg displaying repeats from the *P. falciparum* circumsporozoite protein (PfCSP), has shown moderate level efficacy of modest duration in Phase II/III clinical trials [6], [7], [8] and is progressing toward licensure. The breadth of approaches to protein vaccine design is now continuing to expand as innovative new concepts in next-generation subunit design and antigen discovery [see Doolan *et al.* in this review series] are explored, with the prospects for the development of a highly effective multi-component/multi-stage/multi-antigen formulation seeming ever more likely.

This review will focus on recent progress in protein vaccine design, development and/or clinical testing for a number of leading malaria antigens from the sporozoite-, merozoite- and sexual-stages of the parasite's lifecycle. Progress with PfCSP-based vaccines, especially RTS,S, has been reviewed in detail elsewhere [9], [10] and in this Special Issue [Kaslow et al.], and the development of protein vaccines against the *P. vivax* Duffy-binding protein (PvDBP) are also covered in another article in this review series [Chitnis et al.]. Future prospects and challenges for this field will be summarized.

## 2 Antigen candidates

### 2.1 PfCelTOS

Significant efforts are currently focused on identifying promising antigenic targets expressed on sporozoites and in early liver-stage development with the intent to combine and synergize immune responses against these two stages of the lifecycle. The *P. falciparum* cell traversal protein of ookinetes and sporozoites (PfCelTOS), independently identified by mining genomic [11] and expression [12] sequence databases, is a micronemal secreted-protein. This antigen has been implicated in host cell-traversal and invasion during both motile life-stages of the parasite, thus supporting its candidacy as a pre-erythrocytic target.

Initial studies in mice demonstrated protective efficacy against a heterologous *P. berghei* challenge using a codon-harmonized, *E. coli*-expressed PfCelTOS [13]. These antibodies recognized both *P. falciparum* and *P. berghei* sporozoites, and inhibited gliding motility and hepatocyte invasion *in vitro*. Recently, the contribution of antibodies and T cells as the effectors against parasites at the site of infection was verified using *In vivo* Imaging System (IVIS) [14]. In studies using an experimental clinical-grade adjuvant – a synthetic TLR4 agonist in a stable oil-in-water emulsion designed to enhance humoral and cell-mediated immunity (GLA-SE) – Th1-biased immune responses (with higher IgG2a antibodies, IFN-γ responses and long-lived antibody-secreting cells) were observed in BALB/c mice as compared to mice immunized with the same antigen in Montanide ISA720 [15]. However, when this formulation was tested in a first-in-human clinical trial, while safe and immunogenic, no protective efficacy was observed against controlled human malaria infection (CHMI) (J. Cowden, personal communication). A subsequent Phase Ia trial with PfCelTOS in GSK's AS01B adjuvant is currently ongoing (Clinicaltrials.gov NCT02174978).

Other vaccine strategies have assessed delivery of PfCelTOS using viral vectored vaccines in inbred and outbred mice, however these failed to protect against a challenge with chimeric *P. berghei* parasites expressing the PfCelTOS antigen [16]. Another study sought to combine multiple sporozoite targets, including the PfCelTOS antigen, to broaden humoral and cellular responses. Ferraro et al. evaluated a multi-subunit DNA-electroporation delivery in mice and non-human primates (NHP) [17], and reported plasmid-expressed PfCelTOS antigen-specific antibodies and T cell responses. Recently a chimeric-molecule, composed of three *P. falciparum* 3D7 pre-erythrocytic domains (PfCelTOS; PfCSP-thrombospondin-related-type-1-repeat domain (-TSR); and thrombospondin-related adhesion protein (PfTRAP)-TSR), expressed in *Nicotiana benthamiana* and delivered in Gerbu MM adjuvant induced antibodies in mice that recognized methanol-fixed sporozoites and inhibited invasion of hepatocytes up to 35%, although the specific contribution of PfCelTOS-antibodies was not elucidated [18]. While early indications in preclinical animal studies are encouraging, the potential for efficacy induced by a PfCelTOS-based vaccine in humans is yet to be realized.

### 2.2 PfAMA1

Apical membrane antigen 1 (AMA1) is a micronemal protein that is secreted onto the surface of both merozoites and sporozoites [19], [20], [21]. Historically PfAMA1 has been a leading malaria vaccine candidate (see [22] for a detailed review) and antibodies to AMA1 in multiple *Plasmodium* species block merozoite invasion of RBC and sporozoite invasion of hepatocytes [21], [23]. However, the high number of polymorphisms [24], [25] and *in vitro* data showing a predominantly allele-specific inhibition of anti-PfAMA1 antibodies [26], [27] have dampened expectations. Approaches to vaccine design to try and overcome antigenic heterogeneity are reviewed elsewhere in this series [Plowe et al.].

Despite promising data from NHP studies [28], [29], [30], a key question is whether a PfAMA1-based vaccine can protect humans. This has been evaluated by CHMI with homologous parasites in Phase I/IIa studies using protein- or viral vector-based vaccination strategies [31], [32], [33]. Disappointingly, none of these studies, as well as two recent Phase IIb field trials [34], [35], showed any overall efficacy when using vaccines based on PfAMA1 alone. It should be noted that another study using a DNA prime – adenoviral boost regime reported 27% sterilizing efficacy against mosquito-bite CHMI in healthy US adults when using vectored vaccines encoding PfAMA1 and PfCSP [36]. Cellular immune responses against PfAMA1 were largely associated with protective outcome [36], [37]. Moreover, in one of the field trials of a protein PfAMA1-based vaccine formulated in AS02A adjuvant, 64% efficacy against low frequency, 3D7 vaccine-type parasites, as defined by homology at 9 out of over 60 polymorphic sites, was also observed [35]. This efficacy was not observed when the same population was followed over the next malaria season despite sustained high antibody titers [38].

Lack of efficacy with PfAMA1-based vaccines alone, at least in the CHMI studies, cannot be attributed to polymorphisms in PfAMA1 given that vaccine-homologous parasites were used [31], [32], [33]. Notably the PfAMA1 vaccines have been tested with adjuvants such as AS01B and AS02A, which remain a leading delivery technology in humans for the induction of high antibody concentrations. Consequently, if insufficient antibody titers are the reason that PfAMA1 vaccines failed to protect, then there remains a significant challenge to develop clinically relevant ways of improving the magnitude of anti-PfAMA1-specific antibody [31], [38]. However, another possible reason for the poor efficacy in humans could be due to the vaccines not inducing a threshold concentration of functional antibodies, despite the overall high anti-PfAMA1 titers. Indeed, it remains unknown, for now, what proportion of vaccine-induced PfAMA1 antibodies in humans can provide functional anti-parasitic activity. Recent efforts have largely aimed at enhancing strain-transcendent, neutralizing antibodies by incorporating multiple PfAMA1 alleles or mutagenesis to redirect the immune response toward conserved regions [39], [40], [41], [42], [43], [44], and one ‘diversity covering’ (DiCo) approach has just entered Phase Ia testing in Europe (Clinicaltrials.gov NCT02014727). These approaches increase the repertoire of antibodies against multiple PfAMA1 alleles but do not, however, appear to enhance inhibition of homologous parasites. Therefore developing new approaches to boost the proportion of functional antibodies, and improve the so-called “quality” of the antibody response, against homologous parasites could help advance PfAMA1-based vaccines.

*Plasmodium* merozoite invasion of RBC requires interaction of AMA1 with a conserved 47 amino acid region of rhoptry neck protein 2 (RON2, a parasite protein secreted onto the surface of the RBC), leading to conformational changes in the AMA1 loop regions surrounding the hydrophobic pocket [45], [46], [47]. Although the importance of this interaction has been debated [48], [49], [50], antibodies that bind the AMA1 loops block AMA1–RON2 interaction and inhibit invasion [46], [51], [52]. Interestingly, many of the polymorphisms in PfAMA1 are present in loops surrounding the PfRON2 binding site [25], indicating immune selection and the importance of these antibodies in protection. Since this form of AMA1 in complex with RON2 is functional and possibly exposed on the surface of the merozoite during invasion, a subunit vaccine mimicking this structure could perhaps be better than AMA1 alone. Interestingly, mice vaccinated with the *Plasmodium yoelii* AMA1–RON2L complex, but not PyAMA1 alone, were protected against a virulent *P. yoelii* challenge despite inducing similar levels of anti-PyAMA1 antibodies [53]. Consistent with this, IgG from animals immunized with the complex also inhibited *P. falciparum* homologous parasites significantly more than IgG from animals receiving PfAMA1 alone, suggesting a qualitative shift in the antibody response, *i.e.* a higher proportion of vaccine-induced antibodies that are inhibitory [53]. Indeed, preclinical evaluation in *Aotus* monkeys indicates that vaccination with the complex provides significantly higher protection than PfAMA1 alone against a homologous FVO-strain challenge (PS, LHM, unpublished). These data suggest that a threshold level of functional, neutralizing antibodies could be induced by a PfAMA1–RON2L complex vaccine to confer protection in humans. Efforts are currently underway to assess whether this approach provides protection in humans, and if combining a multi-allele PfAMA1 vaccine in complex with RON2L can protect against both homologous and heterologous parasites. It is tempting to speculate that immune responses to such next-generation PfAMA1-based vaccines may be boosted through natural infections in malaria endemic populations where PfAMA1 is one of the more immunogenic antigens.

### 2.3 PfMSP1

*P. falciparum* merozoite surface protein 1 (PfMSP1), the major surface coat antigen on merozoites, has been extensively investigated as a blood-stage vaccine candidate, primarily based on reports that anti-PfMSP1 antibodies are associated with decreased risk of clinical malaria, albeit, in an allele-specific manner [54]. To date, numerous PfMSP1-based vaccine trials have been completed, with all candidates being considered safe and immunogenic [55]. However, no vaccine candidate comprising of any portion of PfMSP1 has shown either efficacy or reduction of parasite multiplication rates (PMR) following homologous CHMI [33], [56], [57], or following natural infection in Phase IIb field trials [58], [59]. It should be noted that, unlike for the PfAMA1 protein vaccine discussed above, sieve analysis of the PfMSP1 field trial data has not been reported and could provide valuable insight. Such analyses are important in order to document the potential value of a vaccine candidate antigen, whereby a positive outcome then shifts the challenge to approaches for overcoming antigenic diversity.

Early strategies for PfMSP1 focused on antibody induction using subunit protein-in-adjuvant vaccines [59], [60] in heterologous allelic combinations [61] and multi-subunit combinations [62]. More recent clinical strategies have incorporated viral vectored prime-boost regimens to broaden immunity toward functional CD4^+^/CD8^+^ T cell induction [63], and in multi-subunit combinations [33]. In the latter cases, induction of effector T cells was unable to overcome limitations in the induction of effective antibody levels. To further assess PfMSP1-antibody functionality, *in vitro* assays of parasite growth/invasion inhibition activity (GIA/IIA) were performed on immune sera or purified immunoglobulins. In general, modest to low levels of GIA were detected from vaccinated malaria-naïve subjects.

While PfMSP1 vaccine development has largely yielded disappointing outcomes in clinical investigations, several new efforts are focused on alternate expression hosts to improve subunit structure [64], [65]; on forming chimeric molecules to broaden allele-and target-specific immunity [66], [67], [68], [69]; on PfMSP1-nanoparticle delivery platforms [70], [71]; on aggregates and chemical-conjugates [72]; and on viral vectored vaccines in prime-boost regimens co-expressing molecular adjuvants [73]. Despite significant hurdles to their development and evaluation, some limited improvements in PfMSP1-specific antibody functionalities have been reported from animal studies. While these findings may be promising, it remains to be seen whether they are sufficient to exceed the limitations in antibody magnitude, specificity and functionality observed to-date in humans.

### 2.4 PfRH5

In recent years *P. falciparum* reticulocyte-binding protein homolog 5 (PfRH5) has emerged as a promising vaccine candidate antigen against the blood-stage merozoite [74] which, similar to PfMSP1 and PfAMA1, has been assessed using the standardized cell-independent *in vitro* assay of GIA. Antibodies induced by PfRH5 vaccination in preclinical *in vitro* studies have been shown to overcome two long-standing difficulties associated with other merozoite antigens: first, they can block erythrocyte invasion to high efficiency (with lower EC_50_ in terms of antigen-specific antibody concentration than other known antigens [75]); and second, antibodies induced by monovalent vaccines cross-inhibit all *P. falciparum* lines and field isolates tested to-date [74], [75], [76], [77]. Notably these antibodies have been raised by using full-length PfRH5 immunogens, in contrast to earlier work that failed to induce functional antibodies when using fragments of the antigen made in *E. coli* [78], [79]. Generation of full-length PfRH5 protein proved particularly problematic, and thus the earliest promising results were achieved using viral vectored immunization [74], whereby antigen is expressed *in situ* from virally infected muscle cells [80]; also see Ewer et al. in this review series. Since that time, substantial progress has been made with reports of successful full-length PfRH5 protein production from numerous heterologous expression systems including mammalian HEK293 cells [81], *E. coli* [77], [82], baculovirus-infected insect cells [83], [84], wheatgerm [85], and *Drosophila* S2 stable cell lines [86], but notably not yeast-based systems.

Production of PfRH5 antigen in mammalian cells led to the identification of its RBC surface receptor, basigin, with which it forms a critical non-redundant interaction during invasion [87]. The PfRH5 gene is also refractory to genetic deletion [78], [88], confirming the essential nature of its function, however, in the context of natural infection, PfRH5 does not appear to be a dominant target of naturally acquired immune responses [74], [89], [90], [91]. The high degree of PfRH5 sequence conservation is thus associated with low-level natural immune pressure, but also functional constraints linked to basigin binding. Minimal amino acid substitutions in PfRH5 account for loss of basigin binding and/or host RBC tropism (linked to binding basigin orthologues from other species), suggesting the antigen may not easily escape vaccine-induced immune pressure [88], [92], [93].

Most recently high-level efficacy was reported for the first time against a stringent heterologous strain blood-stage challenge in an *in vivo Aotus* monkey-*P. falciparum* challenge model, following vaccination with viral vectors or PfRH5 protein produced from mammalian HEK293 cells [94]. Protection was strongly correlated with anti-PfRH5 serum IgG antibody concentration and *in vitro* functional GIA [94], confirming the utility of this assay to predict *in vivo* protection and for future vaccine candidate down-selection. There is now significant momentum to progress PfRH5-based vaccines into clinical trials. Although the first candidate to enter the clinic is utilizing viral vectored delivery (Clinicaltrials.gov NCT02181088), at least one full-length protein immunogen has entered cGMP production in Oxford, UK using the *Drosophila* S2 cell expression platform [95]. With recent reports of the crystal structure of PfRH5 bound to basigin and neutralizing mouse monoclonal antibodies (mAbs) [84], [86], [96], future developments will likely see rationally designed second-generation protein immunogens seeking to focus antibody induction on critical areas of the molecule. It is likely PfRH5 immunogens will also be developed using various VLP technologies to maximize antibody induction, and the first VLP of bacteriophage MS2 isolated against an inhibitory mAb has already been reported [97]. VLP platforms are also reviewed elsewhere in this series [Wu et al.]. Furthermore, the recent elucidation of a protein complex containing PfRH5 and two binding partners – PfRH5-interacting protein (PfRipr) and the GPI-anchored cysteine-rich protective antigen/PfRH5-PfRipr membrane-anchoring protein (PfCyRPA/PfRRMAP) [98], [99], suggests further opportunities to improve vaccine potency – both antigens elicit functional antibodies alone [82], [98], [99], [100], and IgG responses induced in rats by PfRH5 and PfCyRPA proteins produced in *E. coli* have been shown to synergize [75], [99].

### 2.5 PfSERA5

One family of proteases associated with the merozoite surface and known to be involved in the egress of *P. falciparum* merozoites from blood-stage schizonts is the large multigene family of serine repeat antigens (SERAs) [101], [102]. Of nine members, PfSERA5 is strongly expressed in schizont stages (approximately 0.5–1.5% of the total mRNA) [103] and, together with PfSERA6, is refractory to deletion and essential for parasite growth [104], [105], [106]. PfSERA5-based vaccine candidates were shown to induce antibodies that either protected against blood stage infection *in vivo* [107] or inhibited parasite growth *in vitro* [108], [109]. Recombinant proteins made from the N-terminal domains of PfSERA5 have been shown to be immunogenic and elicited antibodies that inhibited erythrocyte invasion and parasite replication *in vitro* and in animal models; as well as inversely correlate with parasite density and/or malaria symptoms in sero-epidemiological studies in Uganda and Solomon Islands (reviewed in [110]). It is still a challenge to elucidate the exact role(s) of PfSERA5 but selective inhibitors of subtilisin-like serine protease subtilase 1 (PfSUB1), an enzyme involved in the processing of PfSERA5, have been shown to inhibit merozoite egress and parasite maturation [111], [112], [113], [114], [115]. The significance of the tight temporal, yet extensive processing steps of PfSERA5 remain to be fully understood. In some studies, other than proteolysis [116], some regulatory roles (chaperone-like function or substrate recognition and binding) have also been suggested [114], [117]. An immunological “smoke screen” or blocking of the complement pathway is also possible [118], [119].

The SE36 vaccine candidate is a recombinant form of the PfSERA5 N-terminal domain without the polyserine repeats. Expressed in *E. coli*, there are two products under clinical trials: BK-SE36 with aluminum hydroxide gel [120] and a recent iteration, comprising of BK-SE36 and the TLR9 agonist CpG (BK-SE36/CpG) [121]. The BK-SE36 malaria vaccine showed a favorable safety profile in Japanese male adults and in a malaria-exposed population in Uganda aged 6–>20 years old. Local adverse reactions were comparable to other alum-based vaccines: induration, tenderness, pain, swelling, erythema and redness were reported [120]. Immunogenicity analyses indicate that high seroconversion was achieved by vaccination of 1 mL BK-SE36 (equivalent to 100 μg SE36 protein and 1 mg aluminum hydroxide gel) in malaria-naïve healthy Japanese adults and in individuals with low pre-existing anti-SE36 antibody in the malaria-exposed population [120]. Low seroconversion in malaria exposed adults might be interpreted as immunotolerance in this age group, as observed in other clinical trials [122], [123]. Nevertheless, immunogenicity was remarkably high in the 6–10 year old age group in Uganda. In an ancillary analysis from follow-up of the Phase Ib study, vaccination with BK-SE36 was shown to confer over 70% protection against symptomatic malaria for one year [120]. Antibody titer changes and dynamics of malaria infection also suggest that antibody titers against SE36 were boosted after episodes of natural infection, at least for individuals where doubling of antibody titers from the pre-vaccination titer was observed Yagi, Horii et al. (unpublished). Additionally, analysis of *sera5* allele haplotype diversities and haplotype frequencies were similar between parasite isolates from infected vaccinees and the control group (Arisue et al., unpublished data). Importantly, unlike other blood-stage vaccine candidates, the N-terminal regions possessing inhibitory epitopes are intrinsically unstructured [119], thus strict tertiary structure may not be required to elicit protective antibodies. Further studies using the CpG adjuvant to improve the immunogenicity of the antigen is in Phase Ia clinical trial (Horii et al., unpublished), and formal Phase II efficacy testing of the SE36 candidate will be eagerly awaited.

### 2.6 PfGLURP, PfMSP3 and Pfs48/45

Further efforts in the field have focused on the *P. falciparum* merozoite antigens glutamate-rich protein (PfGLURP) and PfMSP3, more recently combined with the gametocyte antigen Pfs48/45. Selection of these target antigens and subunit vaccine design was guided by observations made from studies of naturally acquired immunity (NAI), *in vitro* functional bioassays and assessment in preclinical/clinical studies. Expression of two recombinant subunit vaccine candidates to-date has relied on the use of *Lactococcus lactis* (a generally recognized as safe or ‘GRAS’ organism). These include GMZ2 (a fusion of PfGLURP and PfMSP3) targeting the asexual blood-stage and aimed at reducing the parasite load in order to confer protection against clinical malaria, and more recently Pfs48/45, aimed at targeting the parasite's sexual development within the mosquito and thus seeking to reduce parasite transmission.

GMZ2 is a hybrid protein [124] consisting of conserved domains of the two asexual blood stage antigens from *P. falciparum* – GLURP_27–500_ and MSP3_212–380_ [125], [126], aimed to mimic pathogen components that induce premunition, a state of NAI against *P. falciparum* malaria [127]. The PfGLURP and PfMSP3 components of GMZ2 were chosen to be combined as a hybrid protein based on a series of immuno-epidemiological studies from geographically diverse epidemiological settings [128], [129], [130], [131], [132], [133] with some synergism observed between naturally occurring antibodies against both antigens [132]; functional *in vitro* studies [133], [134]; and population genetic studies of malaria exposed children [135]. Notably the selection of these candidate antigens was prioritized on the basis of the ability of antigen-specific antibodies to elicit antibody-dependent cellular inhibition (ADCI) in conjunction with monocytes, as opposed to cell-independent neutralization in the assay of GIA as used to assess vaccines based on PfMSP1, PfAMA1 and PfRH5. Clinical development of GMZ2 has focused on the use of an Alhydrogel (alum) adsorbed formulation which has proven to be safe and well tolerated in preclinical toxicology studies [136]. This GMZ2/alum formulation has been tested in three Phase Ia/b studies in (i) malaria-naïve German adults [137]; (ii) partially immune Gabonese adults [138]; and (iii) Gabonese children aged 1–5 years [139]. All these trials have shown GMZ2/alum to be safe, well tolerated and immunogenic. Recently, it was demonstrated that the GMZ2/alum formulation elicits antibody titers in humans which are equivalent to those obtained after years of natural exposure [140]. It was therefore decided to test the GMZ2/alum formulation in a multicenter Phase IIb trial in African children to assess safety, tolerability and efficacy in the target population. Results from this trial are awaited.

The Pfs48/45 protein is expressed during the sexual differentiation of the parasite into gametocytes [141] and consists of cysteine-rich domains with multiple disulfide bonds. These constitute distinct conformational B cell epitopes recognized by several mAbs with transmission-blocking activity (TBA) [142]. During their development in the human host, gametocytes remain intra-erythrocytic and are relatively sheltered from the effects of the immune system. However, once inside the mosquito midgut the parasite develops into extra-erythrocytic gametes, exposing Pfs48/45 on their surface, where the antigen can be targeted by antibodies and other components of the blood meal [141]. Naturally acquired anti-Pfs48/45 antibodies are present in endemic populations, and the occurrence of these antibodies is associated with TBA when tested in the standard membrane feeding assay (SMFA) [143], [144], [145], [146]. Overcoming the challenges related to the recreation of the native conformation of Pfs48/45 [147], [148], [149], [150], [151], [152], [153], [154], recently led to the production of the carboxy-terminal 10C-fragment of Pfs48/45 (containing three known epitopes for transmission-blocking antibodies) as a chimera with the N-terminal R0 region of PfGLURP [155]. This resulting chimeric protein elicited broadly inhibiting antibodies against both asexual- and transmission-stages of *P. falciparum*. Further variants with a truncated 10C region are now being explored and show promise in terms of improved yield and induction of functional antibodies in rats [156]. A combination vaccine with antigens from both asexual- and transmission-stages may provide both direct protection against clinical disease and indirect benefit by reducing the spread of the parasite in the population. The future assessment of the merits of such multi-stage subunit approaches in humans will be a key focus of research.

### 2.7 Pfs25

Pfs25, a 25-kDa surface antigen of zygotes and ookinetes is currently the most developed transmission-blocking vaccine (TBV) candidate in the pipeline and the only one that has been tested in human clinical trials [157], along with its ortholog Pvs25 from *P. vivax* [158]. Anti-Pfs25 antibodies induced in humans after vaccination with soluble Pfs25 protein in Montanide ISA51 are functional in the *ex vivo* SMFA and significantly block the development of both laboratory strain and field isolates (from gametocyte donors) of *P. falciparum* [158].

To-date Pfs25 has been successfully produced in a variety of expression systems as a soluble protein unlike the cysteine rich pre-fertilization targets (Pfs48/45 and Pfs230 region C) which have proven more difficult to express in heterologous systems [157]. The TBA of antibodies against Pfs25 usually correlates with the antibody titer, and in humans the absolute concentration of anti-Pfs25 specific IgG required to achieve significant blocking is high (86 μg/mL IC_50_ in the SMFA) [159]. In order to achieve high antibody titers in humans, vaccine candidates will therefore likely require strong chemical adjuvants and/or highly immunogenic delivery platforms. The most potent chemical adjuvants can lead to unacceptable levels of reactogenicity, as reported in the Phase Ia clinical trial of a soluble Pfs25-Montanide ISA51 formulation [158]. Future trials of vaccine candidates with other leading adjuvants in clinical development, such as AS01B or GLA-SE, will hopefully show acceptable reactogenicity profiles.

There has been substantial recent interest in developing improved delivery platforms for Pfs25 protein either by conjugating Pfs25 to carrier proteins or displaying Pfs25 on VLPs. Soluble Pfs25 has been produced in *E. coli* after codon harmonization [160], the wheatgerm cell-free system [161], mammalian HEK293 cells Nikolaeva, Biswas et al. (unpublished), plant-based *N. benthamiana* [162] and *Chlamydomonas reinhardtii* [163], and in yeast (*P. pastoris and S. cerevisiae*) [164], [165]. To-date, protein produced in yeast is the best characterized and the one tested in human clinical trials [158].

Pfs25 has been conjugated to itself [166], NANP repeats of the PfCSP [167], or to carrier proteins like covalent conjugation to outer membrane protein complex (OMPC) of *Neisseria meningitides* [168], exoprotein A of *Pseudomonas aeruginosa* (EPA) [169] and cholera toxin subunit B [170]. Studies in Oxford have recently fused Pfs25 to IMX313, a protein heptamerization technology [73], [171], leading to the expression of a nanoparticle in *P. pastoris* and significantly improved antibody titers and TBA in preclinical studies Lee, Biswas et al. (unpublished). Indeed, most of these efforts have led to significant increase in anti-Pfs25 antibody titer and TBA. In the study where Pfs25 was conjugated to OMPC, the results were highly encouraging as the antibody response in macaques persisted at high-levels for over 18 months [168] – a highly desirable attribute for any vaccine seeking to block malaria transmission for prolonged periods of time. The Pfs25-EPA conjugate formulated with Alhydrogel has now been tested in malaria-naïve adult volunteers in the USA, with extension to Phase Ib field trials in Mali underway. Most recently, a clinical study has begun in the USA to assess a combination of this vaccine with another consisting of EPA conjugated to a region of the gametocyte antigen Pfs230 (Clinicaltrials.gov NCT02334462). The Fraunhofer Institute has also developed a VLP by fusion of Pfs25 to the Alfalfa mosaic virus coat protein, expressed in *N. benthamiana*, which is also currently being tested in humans. The results of these most recent clinical trials of Pfs25-based conjugates are eagerly awaited.

## 3 Concluding remarks

The development of a highly effective vaccine against *Plasmodium* parasites has proved exceptionally challenging, but protein/adjuvant subunits have to-date provided at least one partially efficacious product based on the PfCSP antigen in the form of RTS,S/AS01B [8]. Protein vaccine candidates against other targets have proved disappointing in Phase IIa/b efficacy trials, but the continued need to innovate has driven much progress in terms of antigen design, expression, cGMP production and human delivery. Efficacy results from on-going trials of GMZ2 and SE36 remain eagerly awaited; improved formulations and iterations of PfMSP1, PfAMA1, Pfs25 and PfCelTOS are under active development or in clinical trials; and new antigens such as PfRH5, Pfs230 and Pfs48/45 amongst others are entering cGMP production or Phase Ia clinical testing. Nevertheless, it is likely that a highly effective vaccine seeking to prevent death, disease or transmission, as required by the recently updated Malaria Vaccine Technology Roadmap to 2030 [172], will necessitate new strategies; and key developments likely to be seen in the near future are outlined in Box 1. The next few years promise to be an exciting time as these new candidates, formulations and approaches are assessed in humans.

## Conflict of interest statement

SJD is a named inventor on patent applications covering PfRH5-based vaccines, malaria vectored vaccines and immunization regimes. EA is a named inventor on patent applications relating to PfMSP1- and PfCelTOS-based vaccines. TH holds a patent for BK-SE36. PS and LHM are named inventors on patent applications covering PfAMA1–RON2L complex-based vaccines.

## Disclaimer

“The authors’ views are private and are not to be construed as official policy of the Department of Defense or the U.S. Army. Research was conducted in compliance with the Animal Welfare Act and other federal statutes and regulations relating to animals and experiments involving animals and adheres to principles stated in the Guide for the Care and Use of Laboratory Animals, NRC Publication, 1996 edition.”

## Figures and Tables

**Table 1:** Progress in the clinical development and testing of malaria vaccine candidates comprising recombinant protein/peptide/VLP and adjuvant.

	References
**Recombinant malaria antigens progressed to clinical testing**	
PfCSP; PfTRAP; PfCelTOS; PfAMA1; PfLSA1; PfLSA3; PfMSP1; PfMSP2; PfMSP3; PfGLURP; PfRESA; Pf27A; Pf11.1; PfEBA175; PfSERA5; Pfs230; Pfs25; PvCSP; Pvs25; [PfRH5; VAR2CSA; Pfs48/45; PvDBP].	[173], [174], [175], [176], [177], [178] Clinicaltrials.gov NCT00312702; NCT00509158; NCT01949909; NCT01605786
**Heterologous expression systems used for cGMP manufacture**	
*E. coli; S. cerevisiae; P. pastoris*; *L. lactis*; *N. benthamiana*; *Pseudomonas fluorescens*; *Drosophila* S2 cells.	[179]
**Recombinant antigen delivery platforms tested in clinical trials**	
Soluble protein; LSP; fusion protein; HBsAg VLP; EPA conjugate; Alfalfa mosaic virus coat protein VLP; virosome.	[180]
**Protein adjuvants tested in clinical trials**	
Adjuphos (aluminum phosphate); Alhydrogel (aluminum hydroxide/alum); Alhydrogel + CPG7909; AS01B; AS02A; Montanide ISA720; Montanide ISA51; GLA-SE.	[61], [176]

Antigens, heterologous expression platforms, delivery platforms and adjuvants are listed. Protein-based antigens reported to be in clinical development but not yet in Phase I clinical testing are shown in square parentheses. Exemplar references are only included when information is not provided elsewhere in this review. LSA = liver-stage antigen; RESA = ring-infected erythrocyte surface antigen; EBA-175 = erythrocyte-binding antigen-175 kDa.

## Box 1 Anticipated future developments for protein-based malaria vaccines.

• **Antigen Discovery:** New and promising vaccine candidate antigens from the sporozoite-, merozoite- and sexual-stage parasites will be discovered from numerous approaches making use of “-omic” datasets [5] [Doolan et al. in this review series]. Antigens that are conserved, essential, not necessarily dominant targets of naturally acquired immunity and yet antibody-susceptible would be attractive targets for protein-based vaccines.
• **Antigen Combinations:** These will be explored empirically and rationally, seeking to define combinations of antigens against the same or multiple lifecycle stages that can provide additive, or ideally synergistic, benefit in terms of protective efficacy [75], [181].
• **Structure-based Immunogen Design:** Development of new protein-based immunogens will benefit from structural understanding of how human antibodies successfully block their parasitic targets. Focus may shift from antigen targets *per se* to ‘epitope-based’ targets, with structural biology guiding the design of improved peptide-based vaccines [182] [Patarroyo ME et al. companion paper]; or protein-based vaccines, as being currently explored for viral pathogens such as HIV, influenza and RSV [183], [184].
• **New Protein Expression Platforms for cGMP Production:** It is likely that new heterologous expression systems will be developed to produce new-generation immunogens for malaria, complementing existing capabilities in bacterial- and yeast-based systems. Insect cell platforms have already been progressed for PfRH5 and VAR2CSA [95], and mammalian cell-based platforms have proved exceptionally valuable in preclinical studies for the expression of full-length malaria proteins [81].
• **Improved Protein Vaccine Delivery Platforms:** New generation protein-based vaccines will explore a range of novel delivery platforms seeking to improve antibody immunogenicity in humans. A range of VLP-, particle- or vesicle-based technologies will likely be tested for their ability to deliver arrayed antigen as a highly immunogenic composition. Mixed-modality prime-boost immunization regimes incorporating vectored- and protein-based components will seek to maximize both cellular and humoral immune responses [80], [185], [186]. Improved understanding of B cell and CD4^+^ T follicular helper cell responses [187], [188] could guide the development of vaccine delivery platforms that can enhance the peak and/or longevity of the antibody response.
• **Adjuvants:** On-going development of new vaccine adjuvants, designed on the basis of improved understanding of innate immune sensing mechanisms, will likely be tested with protein-based malaria vaccines seeking high-titer antibody induction.
• **Assay Development and Quantitative Vaccine Assessment:** The on-going development of *in vitro*/*ex vivo* assays and assessment of their relationship to *in vivo* malaria protection will remain an important focus of research. The ability to quantitatively measure antigen-specific antibody concentrations and their related functional activity should allow for the prioritization of new antigens that are inherently more susceptible to antibodies [75], [94], [159], [189], or the definition of immunogens that elicit qualitatively improved IgG responses [53].
• **CHMI Models:** The continued use of CHMI models in Phase IIa clinical trials will allow for rapid efficacy assessment of new protein vaccine candidates and formulations. Vaccines targeting the pre-erythrocytic stages can now be tested using the mosquito-bite or cryopreserved sporozoite (PfSPZ Challenge) models [190], [191]. While the possible effect(s) of blood-stage vaccines on high-level parasitemia cannot be explored in the context of CHMI for obvious safety reasons [192], on-going efforts are aiming to refine the use of the blood-stage CHMI model [193] to assess the impact of new blood-stage vaccines on the parasite multiplication rate (PMR) [194] prior to diagnosis by thick-film microscopy Payne, Draper et al. (unpublished). Other novel approaches using CHMI models, including sub-curative drug treatment regimes, should allow for gametocyte induction and testing of *P. falciparum* transmission-blocking vaccines. The development of cryopreserved sporozoites and blood-stage inocula is now enabling the first modern CHMI studies in malaria-endemic countries [195] and will allow for proof-of-concept vaccine testing in the target population, including individuals with existing degrees of natural immunity. The development of new *P. falciparum* parasite strains for CHMI [196], [197], beyond the historical NF54 strain/3D7 clone, will enable more informative assessment of vaccines against heterologous parasites and more recent isolates; while the establishment of blood-stage inocula for *P. vivax* will allow expansion of efforts into vaccine testing against a second parasite species [198].
